# Effects of monensin supplementation on methane output and behavior of late-lactation Holstein cows

**DOI:** 10.3168/jdsc.2026-1015

**Published:** 2026-03-27

**Authors:** I.M. Toledo, D. Onan-Martinez, M.A.T. de Bari, H. Olmo, G.E. Dahl

**Affiliations:** Department of Animal Sciences, University of Florida, Gainesville, FL 32611

## Abstract

Multiple strategies have been tested in the attempt to reduce enteric methane (CH_4_) emissions and yield from dairy cattle. One of the strategies is supplementation with monensin. Behavioral change strategies are often adopted by dairy cows to adapt to environmental changes. In the present study, 20 cows were supplemented with either 300 mg/cow per day of monensin in 34 g/cow per day of dried distillers grains (MON, n = 10) or only dried distillers grains (CON; n = 10), both top-dressed on TMR. Automated monitoring devices (Nedap, Groenlo, the Netherlands) were used to assess behavioral activity of late-lactation cows supplemented with monensin (MON; n = 5) or nonsupplemented (CON; n = 5). Cows received a leg tag to measure daily lying time, number of steps, and standing bouts and a neck tag to measure eating and rumination time. Behavioral activities were recorded for 21 d before supplementation began and for 28 d during supplementation. Cows were housed in a sand-bedded freestall barn with Calan gates and equipped with a cooling system including shade, fans, and soakers. A GreenFeed system was used to measure enteric gas output. Daily CH_4_ emissions and yield were significantly reduced with monensin. No differences in behavioral activities were observed between MON and CON during the 21-d period before monensin supplementation. During the 28 d of monensin supplementation, daily eating time did not differ between MON and CON. Rumination and lying times were greater in MON compared with CON. Nonsupplemented cows (CON) tended to be more inactive, had more steps per day, and spent more time standing compared with MON cows. No differences between MON and CON cows were observed in standing bouts per day. The present results suggest that monensin reduces daily enteric CH_4_ emissions and yield and monensin supplementation is associated with positive behavioral changes in late-lactation dairy cows.

## Graphical Abstract

**Figure:**
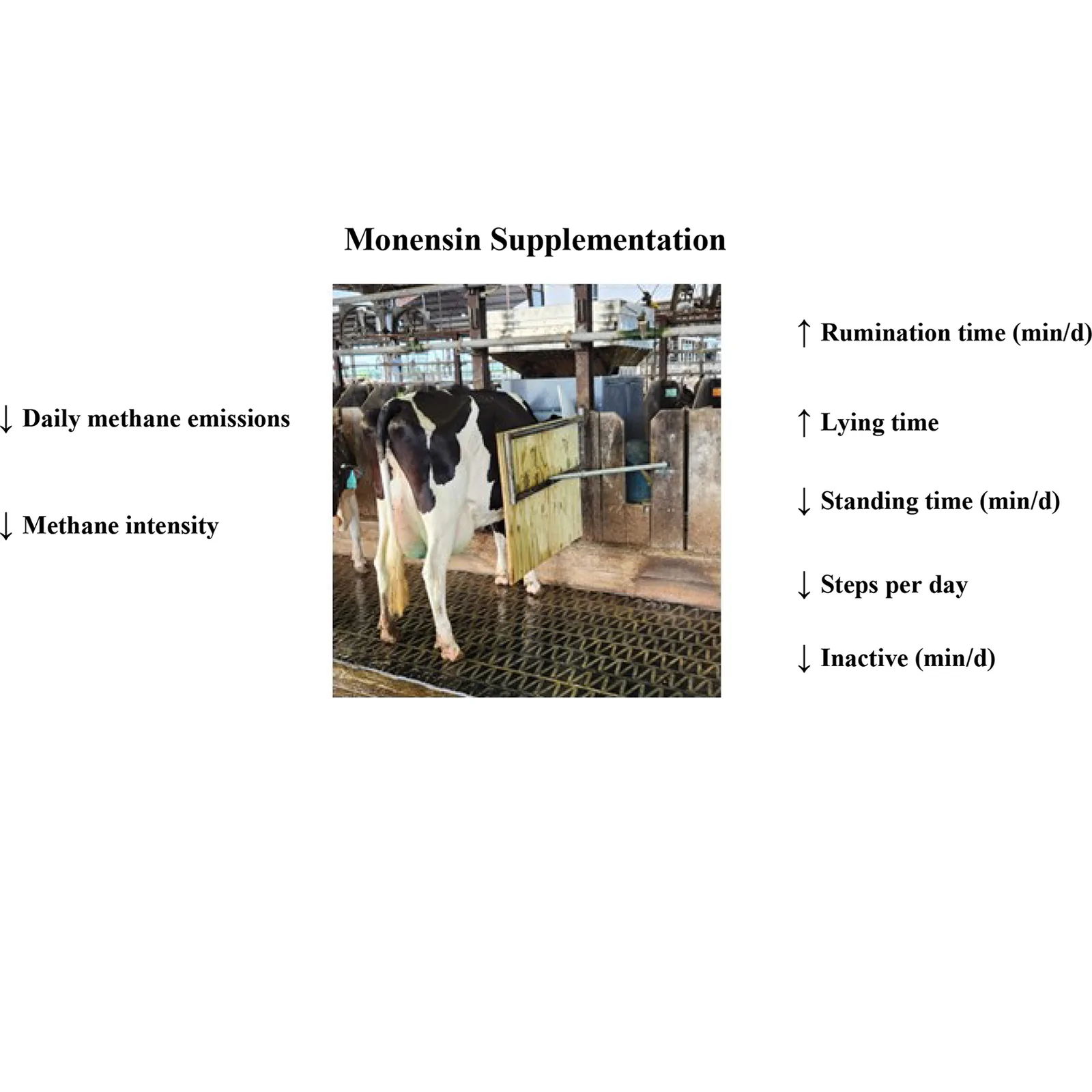

Methane (CH_4_) is one of the major greenhouse gases associated with global warming. In addition to its effects on the environment, ruminal methanogenesis constitutes significant energy loss in the rumen fermentation process, remarkably reducing feed efficiency (Cuervo et al., 2025). Globally, according to the United Nations' Food and Agriculture Organization (FAO), livestock GHG emissions contribute about 12%, whereas in the United States, the Environmental Protection Agency (EPA) in 2016 reported a 9% contribution of CH_4_ from the agriculture sector of which 28.6% comes from the livestock industry.

Methanogenic microorganisms present in the rumen ferment OM and generate CH_4_ by converting carbon dioxide (CO_2_) and hydrogen (H_2_). Efficient feed fermentation and growth of rumen microbiota are supported by these methanogenic microorganisms, which are responsible for keeping low hydrogen levels in the rumen (Matthews et al., 2019). Reducing CH_4_ emissions from cattle could benefit the environment due to their implications on climate change and global warming and enhance cattle feed efficiency to meet the animal protein demand of a growing population (Cabezas-Garcia et al., 2017). One of multiple strategies used to attempt to reduce enteric CH_4_ emissions and yield from dairy cattle is supplementation with monensin, which is an ionophore. Dietary ionophores selectively disrupt ruminal gram-positive bacteria to improve fermentation and reduce the substrate for methanogenic bacteria potentially reducing CH_4_ output (Schären et al., 2017). Variability in CH_4_ emissions while using monensin has been previously associated with stage of lactation, differences in diet composition, level of intake, and product dosage (Odongo et al., 2007; Benchaar, 2016; Rezaei Ahvanooei et al., 2023).

Behavioral strategies adopted by lactating cows to adapt to seasonal and environmental changes include reduced rumination time, decreased DMI, modification of drinking and eating habits, increased standing time, and decreased activity and movement (Wheelock et al., 2010; Soriani et al., 2013; Nordlund et al., 2019; Toledo et al., 2024). Although physiological and behavioral adaptations are necessary, performance and productivity of dairy cows are affected by these changes, which often result in financial losses (St-Pierre et al., 2003; Ferreira et al., 2016). Furthermore, disruption of natural behavior of dairy cows is widely used as welfare and comfort indicators (Haley et al., 2000; Müller and Schrader, 2003) and to investigate animal production parameters (Phillips and Rind, 2001). Due to the potential decrease in CH_4_ output associated with monensin supplementation, and possible changes in behavioral activity particularly in daily rumination patterns to improve feed efficiency, we hypothesized that supplementation with monensin will decrease CH_4_ and positively alter the behavior of late-lactation Holstein dairy cows. The objective of the study was to evaluate the potential positive effect of supplementation of monensin on CH_4_ emission and the behavior of late-lactating dairy cows.

The present study was conducted at the University of Florida Dairy Unit and all procedures were approved by the Institutional Animal Care and Use Committee (IACUC protocol 2022-00000653). A total of 20 late-lactation Holstein cows (i.e., average of 207 ± 13 DIM; ± SE) were enrolled in either a treatment group (**MON**; n = 10), supplemented with 300 mg/cow per day of monensin sodium in 34 g/cow per day of dried distillers grains (**DDG**) or a control group (**CON**; n = 10) receiving DDG alone, both top-dressed daily on the TMR for two 4-wk periods in a crossover arrangement. The cows used in this study consisted of a subset of cows from Onan-Martinez et al. (2026). All cows were housed in a sand-bedded freestall barn equipped with a cooling system including shade, fans, and soakers and individually activated gates for each bunk (Calan Gate System, American Calan, Northwood, NH) to measure individual feed intake. The sand bedding was about 20 cm deep. The dimensions were recommended for a 550- to 700-kg cow. The total stall length was 237 cm and the width was 120 cm, as described by Graves et al. (2010). Cows were fed a ration ad libitum, formulated to meet daily requirements (NASEM, 2021). Due to limited forage availability at the time of the experiment, we fed a diet with concentrate inclusion of 60.3% and 39.7% forage on a DM basis, which is above the typical composition of the regular diet at this farm (usually 45% grain mix and 55% forage). Individual feed intake was calculated by offering feed in the morning and afternoon and measuring feed refusals after 24 h relative to the morning feeding. Full diet details are described in Onan-Martinez et al. (2026).

Cows were milked 3 times per day, and milk yield was recorded throughout the study period using AfiLab milk analyzer (Afimilk Agricultural Cooperative Ltd., Kibbutz, Israel). To measure enteric gas output from individual animals, a GreenFeed System Unit (**GF**; C-Lock Inc., Rapid City, SD) was used. The system consists of automated head chambers that collect short-term measurements of gases from individual cows allowing onsite measurements without altering the housing or normal management. Animals are attracted to the unit by small amounts of bait feed (soyhull pellets) released from a hopper and bait drops are set as needed across the day. The GF unit integrates airflow, gas concentration, and head position measurements during every visit using an extractor fan and sensors allowing for air sample collections from each animal's visit. Air samples pass through an airflow line equipped with a nondispersive infrared sensor for a continuous gas concentration measurement, which is adjusted for air flow rate, temperature, and pressure and corrected for capture rate and background gases. The unit was set to allow the cows up to 9 drops per visit and 6 visits per day with a 3-h interval between visits. All cows were trained to the GF unit for a period of 15 d before the beginning of the trial. Cows were successfully trained, and we obtained a total of 646 valid visits during the length of the experiments with an average of 03:44 min duration per visit. Automated monitoring devices (Nedap, Groenlo, the Netherlands) were used to assess behavioral activity of a subgroup of cows supplemented with monensin (MON; n = 5) or nonsupplemented (CON; n = 5). Cows received a leg tag to measure daily lying time, number of steps, and standing bouts and a neck tag to measure eating and rumination time. Inactivity (i.e., standing rest, lying rest, and sleep) was also measured by the automated monitoring devices. The automated system smart tags have a G-sensor that measures accelerated movements in a particular direction, based on a 3-dimensional accelerometer. The tags distinguish in motions forward and backward, left and right, and up and down. The Nedap automated system has been validated previously by multiple studies (Kooij et al., 2016; Borchers et al., 2021; Quddus et al., 2022). Behavioral activities were recorded for 21 d before supplementation began and for 28 d during monensin supplementation.

The experiment was as a crossover arrangement in a completely randomized design with repeated measures where individual cow was the experimental unit. Data were analyzed using the PROC MIXED procedure of SAS (SAS Institute Inc., Cary, NC). Data were tested for covariance (Levene's test) and normality of distribution was tested by evaluating Shapiro–Wilk statistics using the univariate procedure. The model included the fixed effect of treatment, time (i.e., days), and the interaction between treatment and time. Time was used in the model as a repeated measure. The cow within treatment was used as a random effect. The covariance structure used was the compound symmetry for equal correlation. All statistical comparisons were performed by Tukey–Kramer testing. Significance was set at *P* ≤ 0.05 and tendencies were declared at 0.10 ≤ *P* > 0.05. Sample size was determined by power analysis before the beginning of the study, setting α = 0.05 and power (1 − β) at 0.80. The primary outcomes of interest were eating and rumination time, which were the behavioral outcomes that could be influenced by the monensin supplementation. The study setting and power allowed for a 20% detectable difference for both primary outcomes. The level of variation represented by the SD of 13.5 for eating and 27 for rumination was based on a previous study conducted where the same behavioral activities were evaluated in 34 animals (Toledo et al., 2024).

Results of the present study for daily milk yield, milk composition, CH_4_ intensity relative to ECM, and DMI have been published by Onan-Martinez et al. (2026). Supplementation of monensin (MON) decreased CH_4_ emissions compared with CON (207.1 ± 13.1 vs. 257.2 ± 13.1 g/d, respectively; *P* < 0.01). Methane yield (i.e., grams of CH_4_/kg of DMI) was significantly reduced by MON relative to CON (8.1 ± 0.4 vs. 9.9 ± 0.4 g/kg DMI, respectively; *P* < 0.01). Previous studies (Odongo et al., 2007; Appuhamy et al., 2013) have reported similar results, however differences in CH_4_ reduction may be associated with variations in the diet composition, level of intake, and supplementation dosage differences (Beauchemin et al., 2009; Rezaei Ahvanooei et al., 2023). Additionally, a variability in the effects of monensin supplementation has been associated with the stage of lactation (Rezaei Ahvanooei et al., 2023). When it comes to the effects of supplementation on production, early- and mid-lactation cows seem to benefit the most due to gradual increases in milk production to peak during a state of predominantly negative energy balance, while late-lactation cows are experiencing declining milk output.

Behavioral activity is influenced by various factors including health status (Dittrich et al., 2019), management (Grant and Albright, 2001), season (Toledo et al., 2024), and changes in environmental conditions (Nordlund et al., 2019). In the present study, no differences in behavioral activities were observed between the groups (MON and CON) during the 21-d period before monensin supplementation started. During the 28 d of monensin supplementation, daily eating time did not differ between MON and CON (206.3 ± 6.2 vs. 204.3 ± 6.3 min/d, respectively; *P* = 0.94; Figure 1A); however, rumination was increased in MON compared with CON (538.3 ± 31.1 vs. 432.1 ± 31.1 min/d; *P* = 0.04; Figure 1B). The increase in rumination in the MON group is similar to what has been reported in recent studies (Mikuła et al., 2022; Castaneda et al., 2025), which show that high rumination cows (i.e., increased time/d) had lower enteric CH_4_ emission and intensity compared with cows that spent less time/day ruminating. Increases in rumination (time/d) are associated with shifts in rumen fermentation toward propionate production, leading to a more energy-efficient pathway that uses up more hydrogen, resulting in reduction of CH_4_ precursors and consequent indirect reduction of CH_4_ production (Russell and Strobel, 1989).

**Figure 1:**
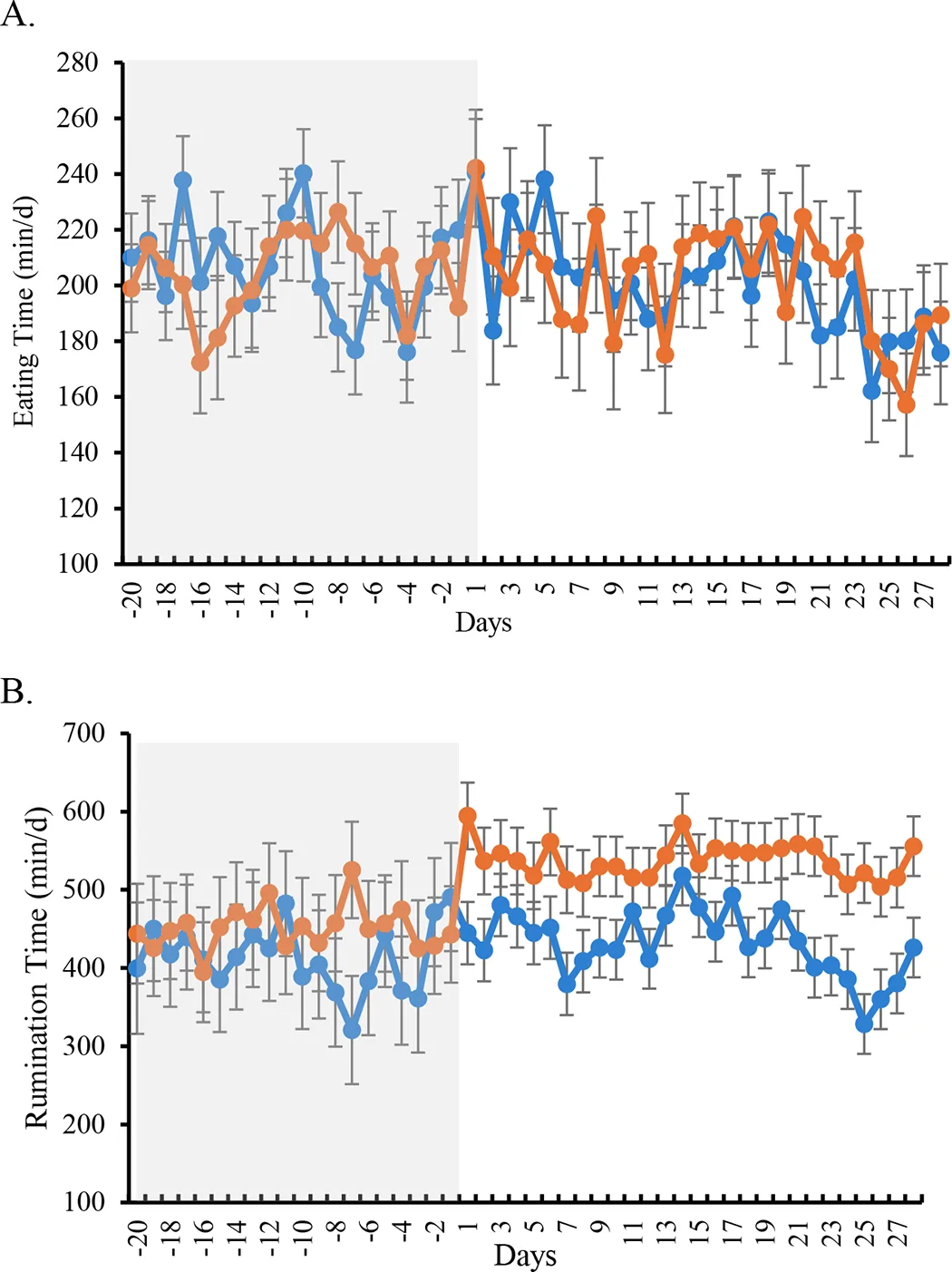
Eating and rumination minutes per day from cows in both supplemented (MON, orange) and control (CON, blue) groups. Daily eating time did not differ between MON and CON (206.3 ± 6.2 vs. 204.3 ± 6.3 min/d, respectively; *P* = 0.94; panel A); however, rumination was increased in MON compared with CON (538.3 ± 31.1 vs. 432.1 ± 31.1 min/d; *P* = 0.04; panel B). Shaded areas represent the period before monensin supplementation. Results are presented as LSM ± SEM.

Lying time per day was greater in MON compared with CON (838.4 ± 26.2 vs. 740.2 ± 27.2 min/d; *P* = 0.03; Figure 2A). Supplemented cows spent less time standing (599.2 ± 26.5 vs. 701.0 ± 26.7 min/d; *P* = 0.02; Figure 2B), tended to be less inactive (686.3 ± 37.0 vs. 790 ± 38.0 min/d; *P* = 0.08; Figure 3A), and had fewer steps per day (2,875 ± 162 vs. 3,675 ± 164 steps/d; *P* < 0.01; Figure 3B) compared with MON cows. No differences between MON and CON cows were observed in standing bouts per day (13.3 ± 1.5 vs. 11.4 ± 1.5.0; *P* = 0.41). Monitoring lying and standing time of dairy cows has been increasingly used as an indicator of welfare (Vasseur et al., 2012). Thus, accommodating and possibly improving dairy cow time budget is essential for their welfare and overall productivity. Essentially, the daily time budget of a dairy cow is the response of a cow to her environment. A healthy and comfortable dairy cow should spend between 12 and 14 h/d lying down (Grant and Albright, 2000). Changes in behavioral routines represent deviations from natural behavior and may affect welfare, health, overall physiology, and production of dairy cows.

**Figure 2:**
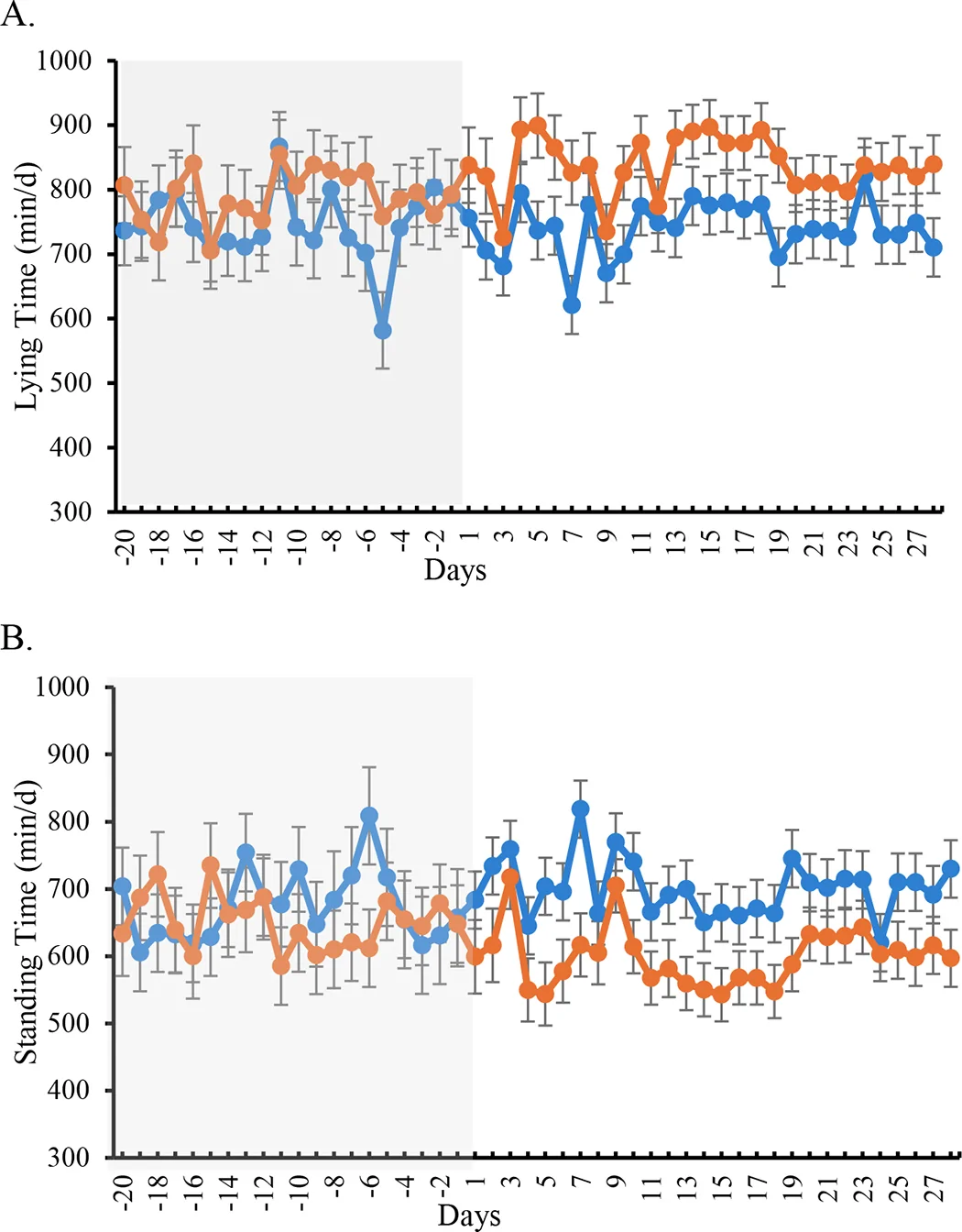
Lying and standing minutes per day from cows in both supplemented (MON, orange) and control (CON, blue) groups. Lying time per day was greater in MON compared with CON (838.4 ± 26.2 vs. 740.2 ± 27.2 min/d; *P* = 0.03; panel A). Supplemented cows spent less time standing (599.2 ± 26.5 vs. 701.0 ± 26.7 min/d; *P* = 0.02; panel B). Shaded areas represent the period before monensin supplementation. Results are presented as LSM ± SEM.

**Figure 3:**
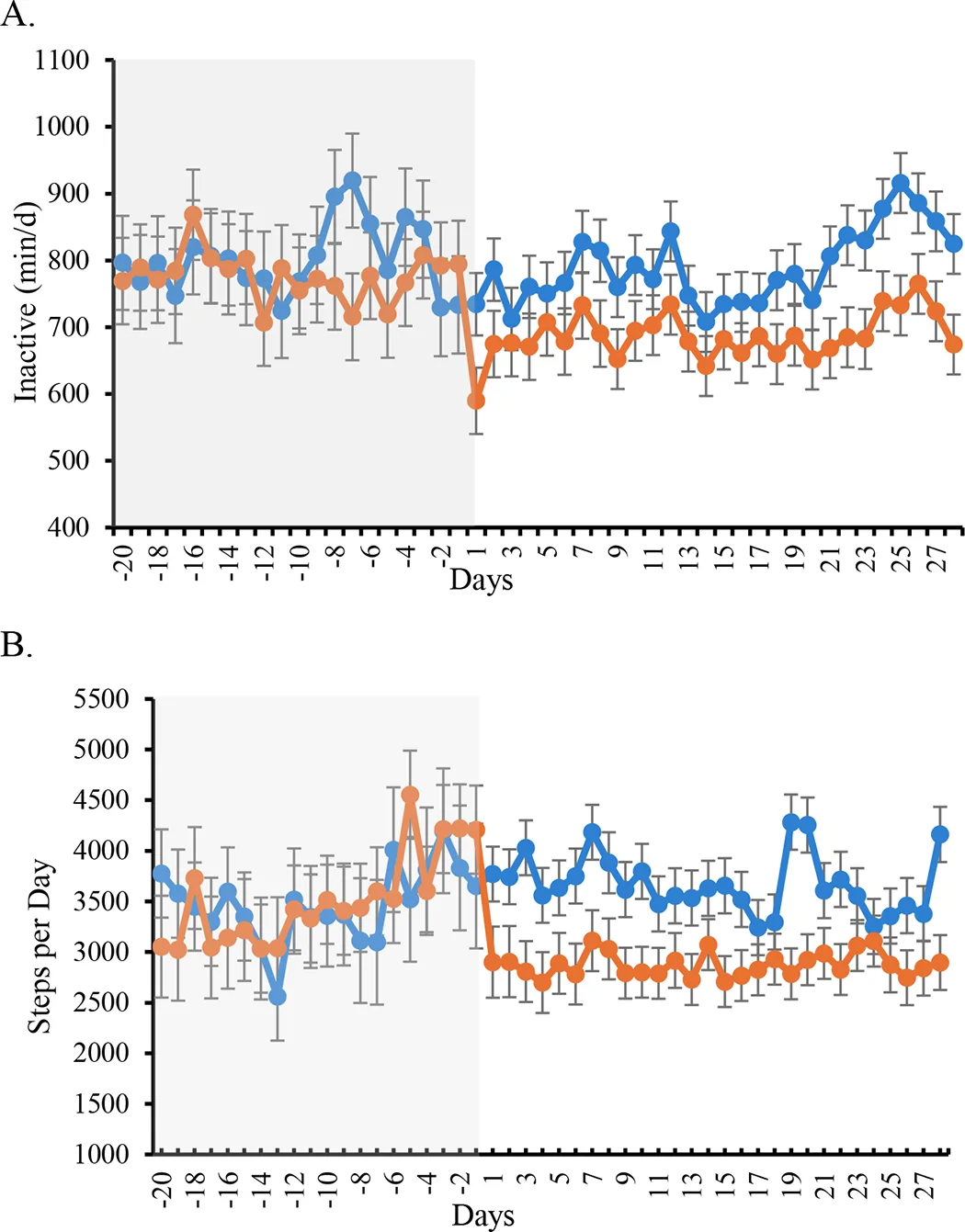
Inactivity (min/d) and steps per day from cows in both supplemented (MON, orange) and control (CON, blue) groups. Supplemented cows tended to be less inactive (686.3 ± 37.0 vs. 790 ± 38.0 min/d; *P* = 0.08; panel A) and had fewer steps per day (2,875 ± 162 vs. 3,675 ± 164 steps/d; *P* < 0.01; panel B) compared with MON cows. Shaded areas represent the period before monensin supplementation. Results are presented as LSM ± SEM.

Our findings support that supplementation with monensin decreases CH_4_ emissions and yield and positively affects late-lactation dairy cow behavior, particularly lying and rumination times, which leads us to conclude that in addition to being used as a potential alternative to lower enteric CH_4_ output and feed efficiency, monensin supplementation may be used as an option to enhance welfare of late-lactating dairy cows.
